# Tramadol and M1 Bioavailability Induced by Metamizole Co-Administration in Donkeys (*Equus asinus*)

**DOI:** 10.3390/ani14060929

**Published:** 2024-03-17

**Authors:** Gabriel Araújo-Silva, Luã B. de Macêdo, Andressa N. Mouta, Maria Gláucia C. de Oliveira, Kathryn N. Arcoverde, Lilian G. S. Solon, José T. Perez-Urizar, Valéria V. de Paula

**Affiliations:** 1 Organic Chemistry and Biochemistry Laboratory, Universidade do Estado do Amapá, UEAP, Avenida Presidente Vargas, 650, Macapá 68900-070, Brazil; gabriel.silva@ueap.edu.br; 2 Department of Animal Science, Universidade Federal Rural do Semi-Árido, 572, Rua Francisco Mota, Presidente Costa e Silva, Mossoró 59625-900, Brazil; luanb.macedo27@gmail.com (L.B.d.M.); andressanmouta@hotmail.com (A.N.M.); glauciacarlos@hotmail.com (M.G.C.d.O.); kathrynnobrega@gmail.com (K.N.A.); 3 Post-Graduate Program in Pharmaceutical Sciences, PPGCF, Universidade Federal do Amapá, UNIFAP, Rod Juscelino Kubitschek, Km2, Macapá 68903-419, Brazil; liliansolon@yahoo.com.br; 4 Facultad de Ciencias Químicas, Universidad Autónoma de San Luis Potosí, 6, Avenida Doutor Manuel Nava, Zona Universitaria, San Luis 78210, Mexico; jpurizar@uaslp.mx

**Keywords:** pharmacokinetic profile, drug interaction, analgesics, metabolites

## Abstract

**Simple Summary:**

The combination of an opioid compound with a nonsteroidal anti-inflammatory drug can lead to additive or enhanced effects while minimizing adverse reactions. Recently, we reported the pharmacokinetic profiles of metamizole and tramadol in donkeys at single doses and without association. However, no studies have reported on the pharmacokinetic profile of the combination of tramadol and metamizole. The objective of this research was to assess pharmacokinetic profile of metamizole co-administered with tramadol at single dose. Behavioral changes in the animals were observed, and at specific intervals, blood samples were taken for subsequent analysis. Analyses were performed using ultra-high-performance liquid chromatography-tandem mass spectrometry. The findings indicate that tramadol and its metabolite presented modified profiles, implying that metamizole and tramadol interact and affect each other’s metabolic processes at the dosages used in this study. Clinical researches are necessary to determine the optimal that effectively addresses the pain relief requirements of the species.

**Abstract:**

Our objective was to assess the pharmacokinetic characteristics of metamizole when administered together with tramadol in a single intravenous dose to donkeys. Ten male animals received 10 mg∙kg^−1^ of dipyrone associated with 2 mg∙kg^−1^ of tramadol (T_2_M_10_) and 25 mg∙kg^−1^ of dipyrone with 2 mg∙kg^−1^ of tramadol (T_2_M_25_). Venous blood samples were taken from groups to determine the pharmacokinetics after drug administration, using initial brief intervals that were followed by extended periods until 48 h. Restlessness and ataxia were observed in two animals in the T_2_M_25_ group. Analysis revealed prolonged detectability of tramadol, 4-methylamine antipyrine, 4-aminoantipyrine (up to 24 h), and O-desmethyltramadol (up to 12 h) after administration. Although metamizole and its metabolites showed no significant pharmacokinetic changes, tramadol and O-desmethyltramadol exhibited altered profiles, likely because of competition for the active sites of CYP450 enzymes. Importantly, the co-administration of metamizole increased the bioavailability of tramadol and O-desmethyltramadol in a dose-dependent manner, highlighting their potential interactions and emphasizing the need for further dose optimization in donkey analgesic therapies. In conclusion, metamizole co-administered with tramadol interferes with metabolism and this interference can change the frequency of drug administration and its analgesic efficacy.

## 1. Introduction

Donkeys are animals primarily used for manual labor in developing countries, where they transport materials and people in challenging conditions such as extreme heat and humidity, noise, and urban pollution. They are often exposed to the risk of accidents owing to heavy traffic [1,2]. These factors make donkeys more susceptible to trauma and other pathologies that can result in pain and increased stress levels.

Metamizole (MET), commonly known as dipyrone, is often categorized as a pyrazolone derivative, functioning as a non-opioid analgesic, and is of particular importance to veterinarians in the field of equine care because it presents good analgesic effects and excellent antipyretic properties, and is recommended for managing colic syndromes, alleviating muscle pain and fever, and recovery after surgical interventions [3,4]. However, it was withdrawn from markets in the United States, Japan, Iran, and the United Kingdom because of its rare side effect in humans, a hematological condition called agranulocytosis [5]. Metamizole is rapidly hydrolyzed to its metabolite 4-methylaminoantipyrine (MAA) and its by-product, 4-aminoantipyrine (AA). The pharmacological effects are attributed to MAA, which is formed in much larger amounts than other smaller metabolites are [6].

Tramadol is an opioid analgesic that is widely prescribed by veterinarians [7]. It is routinely used to treat acute and chronic pain in animals [8,9]. Pharmacokinetic research on tramadol and its primary metabolite, O-desmethyltramadol (M1), has highlighted interspecies variations in drug metabolism, underscoring the importance of conducting pharmacokinetic studies to establish appropriate dosage schedules for various species [10,11,12].

The concurrent administration of an opioid and a pyrazolone derivative NSAID can yield synergistic benefits, enhancing the therapeutic effects while mitigating the adverse reactions associated with each medication [13]. In a previous study, 25 combinations of different doses of the combination of metamizole (56.2–562.3 mg∙kg^−1^) and tramadol (3.2–56.2 mg∙kg^−1^) were evaluated through a single administration, with each combination assessed for additive or potentiated anti-nociceptive effect in mice when compared to the effects of treatment using singular drugs [14].

Research on the pharmacology of numerous drugs in donkeys remains limited, highlighting a significant gap in the existing literature. The drugs used to treat these animals have been frequently developed and recommended for horses [15]. Donkeys differ from horses in behavioral, physiological, and pharmacological aspects [16]. Therefore, knowledge of the pharmacokinetic properties of drugs is fundamental to guarantee effective and safe therapeutic administration [7].

This study aimed to evaluate the pharmacokinetic profile of a single intravenous dose of metamizole co-administered with tramadol in donkeys. We hypothesized that metamizole and tramadol could compete for the same enzymes, causing changes in the concentrations of metabolites of both metamizole and tramadol.

## 2. Materials and Methods

### 2.1. Animals and Experimental Design

This study was approved by the Institutional Animal Use Ethics Committee of Universidade Federal Rural do Semi-Árido (Approval number 23091.006896/2019-47). For this study, ten adult male northeastern Brazilian donkeys aged between 2 to 14 years (mean age of 6.4 ± 3.1 years), with weights ranging from 110 to 145 kg (average weight 126 ± 11.8 kg), were selected. These donkeys were sourced from the Apodi Animal Protection Association (APA). Eligibility for participation in the study necessitated that the donkeys were deemed to be in apparent good health as determined by comprehensive physical and laboratory assessments. Physical examination was based on cardiorespiratory and abdominal auscultation, capillary refill time, mucous membrane color, and fecal characteristics. Laboratory examinations included hemograms and urea, creatinine, alanine aminotransferase, aspartate aminotransferase, and total protein plasma concentrations.

Four weeks before the study began, the animals were treated with an oral dose of ivermectin, 1 g per 100 kg of body weight (Piraverme^®^ Lavizoo, Registro, Brazil) for deworming and vaccinated against rabies using (Rai-Vet Líquida^®^, Vaxxinova, São Paulo, Brazil). They were accommodated in groups of four within an outdoor enclosure measuring 17 m by 13 m, equipped with shaded areas. The diet for the donkeys consisted of 7.5 kg of Napier grass (*Pennisetum purpureum*) per 100 kg body weight and 1.1 kg of a concentrate mix (comprising ground corn, soybean, wheat bran, common salt, and calcitic limestone) provided twice daily, alongside unlimited access to water. A four-week acclimatization period was allotted for the donkeys to adjust to their new surroundings and human handling.

The day before starting the treatment, the animals were moved to individual stalls, where they were fasted for 10 h and water was withheld for six hours, respectively. Following this preparatory phase, the animals underwent two distinct treatments: in the T_2_M_10_ treatment, the ten donkeys received 10 mg∙kg^−1^ of metamizole (D-500^®^, Zoetis, São Paulo, Brazil, 500 mg/mL) associated with 2 mg∙kg^−1^ tramadol (Tramadon^®^, Cristália—Produtos Químicos Farmacêuticos Ltda, São Paulo, Brazil, 50 mg/mL), whereas in the T_2_M_25_ treatment, the same ten animals received 25 mg∙kg^−1^ of metamizole (D-500^®^, Zoetis, São Paulo, Brazil, 500 mg/mL) associated with 2 mg∙kg^−1^ tramadol (Tramadon^®^, Cristália Produtos Químicos Farmacêuticos Ltda, São Paulo, Brazil, 50 mg/mL), in each case delivered separately, one in each jugular vein, in a double administration of 10 mL (metamizole) and 20 mL (tramadol) made with 0.9% NaCl solution. For the intravenous (IV) administration of metamizole and tramadol, as well as for the collection of blood samples, the administration of the drugs was consistently carried out by the same individual. For the purposes of infusion, thorough antisepsis was conducted similar to surgical preparations, including skin cleaning, followed by the placement of a 16G caliber catheter attached to a 3-way tap, which was then securely inserted into both the animals’ jugular veins. The administration of the drugs was achieved using two infusion pumps (Syringe Pump ST670^®^, Samtronic, São Paulo, Brazil) over a span of two minutes. Following administration, the animals were monitored for signs of adverse effects such as ataxia, restlessness, salivation, sweating, and muscle spasms. The occurrence and duration of these effects were evaluated. One hour after the drug administration, water was made available to the animals, and food was offered at regular intervals thereafter.

Blood samples, each amounting to 10 mL, were drawn at specific intervals: immediately prior to the administration of the drug (0 or baseline); at 5, 10, 20, 30, 40, and 50 min; and then at 1.0, 1.15, 1.3, 1.45, 2.0, 2.5, 3.0, 4.0, 6.0, 8.0, 12.0, 24.0, and 48.0 h following the administration. These samples were promptly placed into tubes containing ethylenediaminetetraacetic acid (EDTA). Within 30 min of collection, plasma was extracted by centrifuging the samples at 1715× *g* for 10 min at ambient temperature. The separated plasma was then frozen and stored at −80 °C in cryogenic vials until further analysis.

### 2.2. Sample Extraction Procedure

The aliquots of the plasma samples (250 μL) were supplemented with 10 μL of 0.1 mg/mL metoprolol solution (internal standard) and 800 μL of acetonitrile, followed by vortex homogenization for 60 s, and then the samples were centrifuged for 5 min at 14,200 rpm. The supernatant (900 µL) was transferred to vials for injection in the chromatographic equipment.

### 2.3. Instrumentation and LC and MS Conditions

For the chromatographic analysis, the ultra-performance liquid chromatography system, which was coupled with mass spectrometry (UHPLC-MS/MS) system was used, consisting of Nexera 2 UHPLC coupled to an LCMS-8040 mass spectrometry detector (Shimadzu^®^, Kyoto, Japan) and BEH C18 column (1.7 μm, 2.1 × 75 mm) (Shimadzu^®^, Kyoto, Japan). The mobile phase consisted of acetonitrile and 0.1% formic acid solution (75:25, *v*/*v*) at 0.3 mL/min. The running time was 2.0 min; the volume of sample injection was 5.0 μL. The column temperature was adjusted to 40 °C and the automatic sampler refrigerator was set to 5 °C. For tramadol, M1, MAA, and AA, the mass spectrometer was adjusted in the multiple reaction monitoring (MRM) mode, utilizing positive electrospray ionization (ESI). The collision energy and cone voltage were 12 V and 19 V, respectively. The flow rates of the cone gas and desolvation were calibrated to 150 L/min and 600 L/min, respectively, using Argon as the collision gas at a flow rate of 0.15 mL/min. A mass spectrometer was configured to monitor the transitions of the main ion and fragment ion ranges, the mass and mass-to-charge ratio (*m*/*z*) of the main ions for tramadol, M1, metoprolol, MAA and AA was 264.0, 250.0, 268.1, 218.20, 204.20, respectively, whereas the corresponding transitions for the fragment ions were 264.0 > 58.0, 250.0 > 58.0, 268.1 > 131.1, 218.20 > 159.10, 204.20 > 76.90. With a residence time of 0.3 s. MRM data were acquired and analyzed using the Labsolution software 6.9 (Shimadzu^®^, Kyoto, Japan).

### 2.4. Validation

The analytical method was validated according to the criteria established by the International Conference on Harmonization [17] and the Brazilian National Health Surveillance Agency (ANVISA) in Resolution of the Collegiate Board (RDC) number 166/2017 [18]. The following factors were evaluated: linearity (MAA and AA: 800–40,000 ng/mL; Tramadol and M1: 5–5000 ng/mL; metoprolol: 1000 ng/mL), repeatability, reproducibility, and selectivity in solution and plasma; stability by short- and long-term methods; freezing and thawing cycles with lower and upper limit control samples; and controls at low, medium, and high concentrations (MAA and AA: 2400; 12,000 and 30,400 ng/mL; Tramadol and M1: 15; 1000 and 3750 ng/mL). Standard drug solutions were added to drug-free plasma to create a calibration curve. In addition, quality control (QC) samples were prepared, which were utilized to assess absolute recovery, accuracy, and both intra- and interday precision. The selectivity of the method was evaluated by establishing the lower limit of quantitation (LLOQ) using drug-free plasma. The limit of detection (LOD) and lower limit of quantification were evaluated based on the signal-to-noise ratio (SNR) of three replicates of blank specimens fortified with decreasing quantities of each compound. LOD and LLOQ (metamizole: 840 ng/mL and tramadol: 5.25 ng/mL). Stability (long term in biological matrix at −70 °C; bench temperature at room temperature (20 °C); three freeze–thaw cycles and samples processed in the automatic sampler) was also evaluated.

### 2.5. Pharmacokinetic Analysis and Statistics

Pharmacokinetic profiles were obtained using a non-compartmental analysis with WinNonlin 6.2.1 software (Pharsight, Mountain View, CA, USA, 2011). The observed variables were: the maximum plasma concentration (Cmax), extrapolated concentration without time 0 (C_0_), time to reach Cmax (Tmax), area under the plasma concentration curve from time zero until the moment of the last measurable concentration (AUC_0 → t_), and the extrapolation of the AUC to infinity (AUC0 → ∞), volume of distribution (Vz), clearance (Cl), elimination half-life (T_1/2_); mean residual time until the last measurement (MRT0 → t), and mean residual time from zero to infinity (MRT0 → ∞).

Statistical analyses were performed using BioEstat^®^ version 5.0,(Instituto Mamirauá, Belém, Brazil). The normality of all parameters was analyzed using the Shapiro–Wilk test. Except for Tmax which was evaluated using the Mann–Whitney test and expressed as a median, all other parameters were compared using the T test and expressed as mean and standard deviation. Differences were considered statistically significant at *p* < 0.05.

## 3. Results

After optimizing the chromatographic parameters and defining the analytical variables, the method was validated to ensure data accuracy. All calibration curves showed correlation coefficient (R^2^) values greater than 0.99, demonstrating the linearity between the equipment responses and curve concentrations. This method showed good selectivity, reproducibility, and repeatability, with a relative standard deviation of less than 5%. Moreover, the samples were stable under the conditions used for analysis.

**Figure 1:**
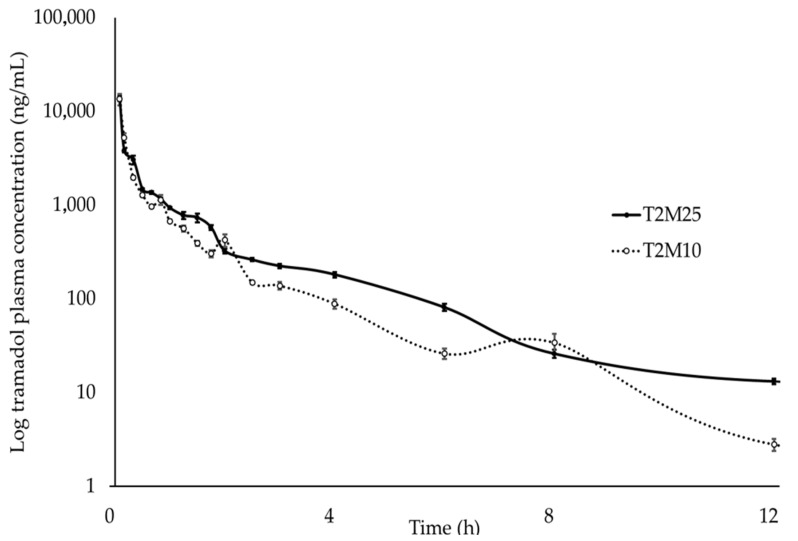
Log_10_ plasma concentration vs. time for tramadol following intravenous administration of metamizole (25 mg∙kg^−1^ and 10 mg∙kg^−1^) and tramadol (2 mg∙kg^−1^) in ten healthy northeastern Brazilian donkeys. Notes: Black circles (•) represent the mean and standard deviation results of the group treated with 2 mg∙kg^−1^ tramadol and 25 mg∙kg^−1^ metamizole; white circles (◦) indicate the mean and standard deviation of the group treated with 2 mg∙kg^−1^ tramadol and 10 mg∙kg^−1^ metamizole.

**Figure 2:**
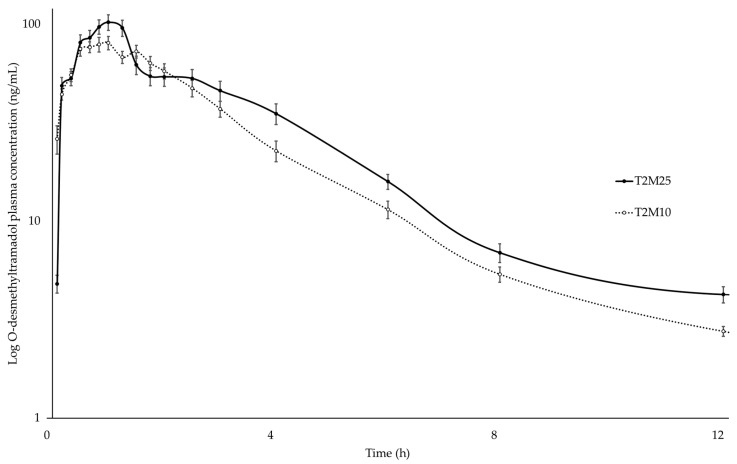
Log_10_ plasma concentration vs. time for O-desmethyltramadol (M1) following intravenous administration of metamizole (25 mg∙kg^−1^ and 10 mg∙kg^−1^) and tramadol (2 mg∙kg^−1^) in ten healthy northeastern Brazilian donkeys. Notes: Black circles (•) represent the mean and standard deviation results of the group treated with 2 mg∙kg^−1^ tramadol and 25 mg∙kg^−1^ metamizole; white circles (◦) indicate the mean and standard deviation of the group treated with 2 mg∙kg^−1^ tramadol and 10 mg∙kg^−1^ metamizole.

**Figure 3:**
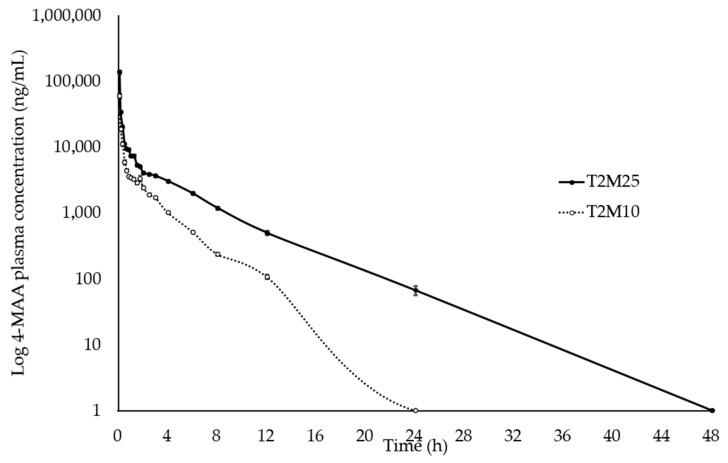
Log_10_ plasma concentration vs. time for N-methyl-4-aminoanthypyrine (MAA) following intravenous administration of metamizole (25 mg∙kg^−1^ and 10 mg∙kg^−1^) and tramadol (2 mg∙kg^−1^) in ten healthy northeastern Brazilian donkeys. Notes: Black circles (•) represent the mean and standard deviation results of the group treated with 2 mg∙kg^−1^ tramadol and 25 mg∙kg^−1^ metamizole; white circles (◦) indicate the mean and standard deviation of the group treated with 2 mg∙kg^−1^ tramadol and 10 mg∙kg^−1^ metamizole.

**Figure 4:**
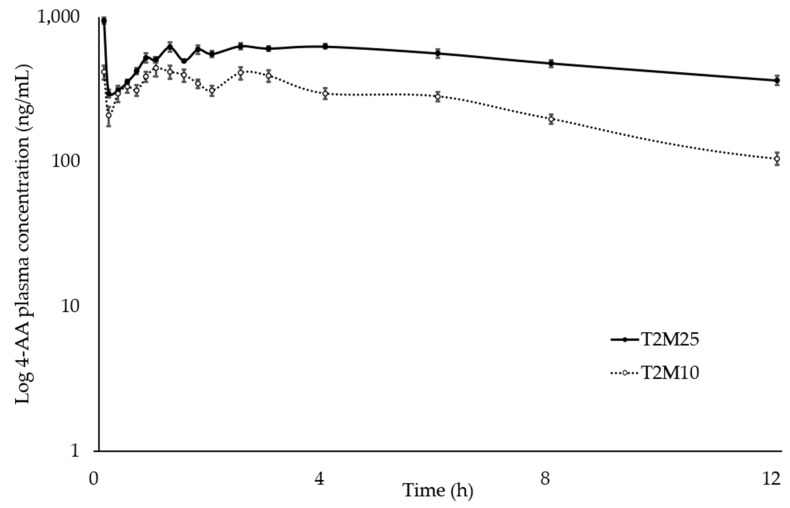
Log_10_ plasma concentration vs. time for 4-aminoanthypyrine (AA) following intravenous administration of metamizole (25 mg∙kg^−1^ and 10 mg∙kg^−1^) and tramadol (2 mg∙kg^−1^) in ten healthy northeastern Brazilian donkeys. Notes: Black circles (•) represent the mean and standard deviation results of the group treated with 2 mg∙kg^−1^ tramadol and 25 mg∙kg^−1^ metamizole; white circles (◦) indicate the mean and standard deviation of the group treated with 2 mg∙kg^−1^ tramadol and 10 mg∙kg^−1^ metamizole.

The mean plasma concentrations of tramadol, M1, MAA, and AA were plotted on a comparative chart of elapsed time (Figure 1, Figure 2, Figure 3 and Figure 4).

After intravenous administration, tramadol, MAA, and AA were detected during 24 h of analysis, and M1 during 12 h.

The pharmacokinetic parameters obtained for tramadol, M1, MAA and AA after intravenous administration of metamizole at doses of 10 mg∙kg^−1^ or 25 mg∙kg^−1^ in association with tramadol (2 mg∙kg^−1^) are shown in Table 1, Table 2, Table 3 and Table 4, respectively. Additionally, for analysis, the obtained data were compared with results from previous studies conducted by the same research group.

Analyzing Tramadol, it was observed that AUC0 → ∞ and Cl were higher for T_2_M_10_ than for T_2_M_25_, while T_1/2_; MRT0 → ∞; MRT0 → t were significantly higher in the T_2_M_25_ group. Regarding its metabolite, M1, T_1/2_; MRT0 → ∞; MRT0 → t were significantly higher for T_2_M_25_.

Regarding the Vz, MRT0 → ∞, T_1/2_ of MAA and MRT0 → t of AA varied significantly between groups, being higher in animals that received 25 mg∙kg^−1^ of metamizole.

Adverse effects were noted after the intravenous administration of metamizole and tramadol. Restlessness and ataxia were observed in two animals in the T_2_M_25_ group but not in the T_2_M_10_ group.

## 4. Discussion

In this study, the evaluation of the pharmacokinetic profile of two doses of metamizole in co-administration with 2 mg∙kg^−1^ of tramadol in donkeys were investigated.

The combination of metamizole and tramadol has been used to treat moderate-to-severe pain in animals affected by neoplasms or arthritis, or undergoing castration surgery [21,22,23]. The results showed that this association has the potential to improve antinociceptive effects [14]. However, these studies were conducted on small animals or laboratory animals.

In the realm of veterinary medicine, donkeys frequently receive therapeutics prescribed according to dosages and intervals recommended for horses, primarily due to the scant availability of donkey-specific drug labeling guidelines [24]. This study marks a pioneering effort to investigate the pharmacokinetic profiles of tramadol and metamizole specifically in donkeys. The findings from this research could serve as a foundational scientific basis for subsequent pharmacodynamic investigations and clinical trials aimed at optimizing pain management strategies for this species.

Metamizole is rapidly hydrolyzed into its two metabolites (MAA and AA) [25] and therefore, it was not possible to obtain the minimum concentrations for quantification at predetermined times, making it impossible to construct the pharmacokinetic profile of this prodrug. Therefore, its metabolites (MAA and AA) are used as markers for pharmacokinetic studies of this drug [6]. Tramadol is a drug carried to the liver, where it is metabolized in M1 and can be measured using the method employed.

The metabolites 4-methylaminoantipyrine and O-desmethyltramadol, the active metabolites of metamizole and tramadol, respectively, have more potent analgesic activity than those of their parent drugs [4]. Since both drugs are metabolized by the enzyme CYP3A4, increasing the metamizole dose from 10 to 25 mg∙kg^−1^ extends their blood presence. This is shown by the marked increase in MRT and T_1/2_ in groups treated with 25 mg∙kg^−1^ of metamizole and in donkeys administered 2.5 mg∙kg^−1^ of tramadol intravenously, suggesting the combination could boost their therapeutic effects [4,26,27,28].

Tramadol clearance was higher in the animals that received 10 mg∙kg^−1^ of metamizole. Both drugs are metabolized by the same enzymes. These results suggest that increasing the dose of metamizole promotes competition for the CYP3A4 binding site, reducing the metabolism of tramadol and prolonging its elimination.

Tramadol exhibits a half-life of 0.97 h at the dosage used in the present study and 1.48 h at a dose of 4 mg∙kg^−1^; however, these data were obtained when tramadol was used alone [19]. In another study, similar values were found, and a half-life of 1.55 h was obtained in donkeys, wherein the drug was rapidly metabolized to N-desmethyltramadol, an inactive metabolite which contributed to the drug being less effective in this species than in others [26]. In the present study, concomitant administration of metamizole increased the half-life of tramadol to 8.12 h (10 mg∙kg^−1^ metamizole) and 13.62 h (25 mg∙kg^−1^), as well as that for M1 (an active metabolite in animals).

The combination of these drugs possibly increases the clinical efficacy of tramadol in donkeys. However, clinical trials are required to determine whether this increased half-life is useful for pain management. An increase in the metamizole dose promoted an increase in the half-lives of tramadol and O-desmethyltramadol. These three substances are metabolized in the liver under the action of cytochrome P450 enzyme variation 3A4, causing it to become overloaded and thus reducing the speed of metabolization of these drugs. However, this half-life extension cannot definitively be deemed beneficial from a clinical point of view. The continuous administration of tramadol and metamizole in rats subjected to the hot plate test initially promoted an improvement in the response; however, with subsequent doses, its efficiency reduced by up to 40%, probably due to the increased opioid tolerance in the animal [14].

Regarding the pharmacokinetic parameters of MAA in donkeys, MRT was higher in animals that received 25 mg∙kg^−1^ of metamizole. The authors of [28] used metamizole in horses and observed an MRT of 3.70 h. This was similar to our data at the same dose and suggests that, regardless of whether it was used alone, the duration of the effect of metamizole did not vary. In addition, the half-life of MAA presented increased with a dose of 25 mg∙kg^−1^. Giorgi et al. [29] reported a half-life of 3.34 h, which differs from our study, which found 4.51 h. This variation may be associated with competition for the active site of the CYP3A4 enzyme, suggesting an enzymatic interaction in which tramadol and MAA compete for the same metabolic pathway [30].

Stewart et al. (2011) [31], in their study on the pharmacokinetics and adverse effects of tramadol when administered intravenously to horses, noted that 7 out of the 12 animals experienced muscle fasciculations within three minutes of receiving a 5 mg∙kg^−1^ dose. Meanwhile, another research [19] focusing on the intravenous application of tramadol in donkeys found adverse reactions in just one animal at a 2 mg∙kg^−1^ dosage and in 7 animals when the dosage was increased to 4 mg∙kg^−1^.

However, no adverse reactions were observed in donkeys treated with tramadol and in mice treated with tramadol and metamizole [13,26]. The reactions found in the two donkeys that received metamizole and tramadol (T_2_M_25_) were probably due to the speed or volume of administration. The Cmax of tramadol and M1 did not differ between the groups treated with 10 mg∙kg^−1^ or 25 mg∙kg^−1^ of metamizole. Moreover, the intravenous administration of drugs in large volume and at a fast rate demonstrably causes adverse effects, mainly, of neurological order [31,32], as reported in our study.

The analgesic efficacy and side effects induced by both tramadol and metamizole vary among different species, influenced not only by intrinsic hepatic metabolism but also by genetic polymorphisms within the CYP450 subfamilies [33]. For instance, while the impact of tramadol can be significantly altered by the efficiency and quantity of a specific CYP450 enzyme in an individual, metamizole, known for its analgesic and antipyretic properties, may also have its efficacy and side effect profile modified by similar genetic and metabolic factors. These variations in CYP450 phenotypes affect the metabolism, accumulation, or elimination rates of both substances, directly influencing the success or failure of analgesic outcomes and the potential for adverse effects [14]. According to Ruel and Steagall (2019) [34], some medical centers are now integrating computerized clinical decision support systems that include pharmacogenomics tools to customize treatment with tramadol and metamizole. This strategy is based on individual pharmacogenomic profiles (e.g., extensive, intermediate, or poor metabolizers) to predict the safety and efficacy of the combined or individual therapy of these drugs.

The combination of drugs can lead to complex interactions that affect their metabolism, potentially resulting in increased plasma concentrations and bioavailability, which can lead to larger drug distribution volumes and adverse effects. This phenomenon occurs when drugs compete for the same metabolic pathways, especially those involving cytochrome P450 enzymes, leading to slower metabolism and a prolonged presence in the plasma of the drug and its metabolites. Such interactions highlight the importance of understanding the pharmacokinetic profiles of drugs when used in combination [35].

As this study aimed to evaluate the pharmacokinetic profile of analgesic drugs widely used in companion species, it is only possible to determine whether there is an interaction within the scope of the metabolism of these drugs, and we could not infer which of the two associations is more beneficial in the treatment of pain. Further clinical studies are needed to resolve these issues in northeastern Brazilian donkeys.

## 5. Conclusions

Pharmacokinetic research on donkeys is limited, making this pioneering study on the drug interactions between tramadol and metamizole in donkeys significant. It indicates that these drugs may impact each other’s metabolism. Further clinical research is essential to determine the optimal dosages for effective analgesia in donkeys, reducing the need to rely on dosages extrapolated from horse studies.

## Figures and Tables

**Table 1:** Pharmacokinetic parameters of tramadol (2 mg∙kg^−1^) in association with metamizole (10 mg∙kg^−1^ or 25 mg∙kg^−1^) intravenous (IV) administration in ten donkeys.

Tramadol	(Tramadol 2 mg∙kg^−1^ IV) Mouta et al., 2021 [19]	T_2_M_10_ (Tramadol 2 mg∙kg^−1^ and Metamizole 10 mg∙kg^−1^ IV)	T_2_M_25_ (Tramadol 2 mg∙kg^−1^ and Metamizole 25 mg∙kg^−1^ IV)
C_0_ (ng/mL)	6150 ± 1717 ^b^	9995 ± 2095 ^a^	13,776 ± 3254 ^a^
AUC0 → ∞ (h.ng/mL)	2663 ± 1828 ^b^	3882 ± 764 ^a^	5008 ± 808 ^b^
T_1/2_ (h)	0.97 ± 0.17 ^a^	8.12 ± 1.55 ^b^	13.62 ± 1.26 ^a^
Vz (L/h/kg)	1.32 ± 0.63 ^a^	9.15 ± 7.01 ^b^	8.75 ± 7.21 ^b^
Cl (L/h/kg)	1.01 ± 0.50 ^a^	0.76 ± 0.32 ^b^	0.42 ± 0.18 ^c^
MRT0 → ∞ (h)	1.34 ± 0.36 ^a^	2.10 ± 0.48 ^b^	8.35 ± 2.44 ^c^

Notes: Results are presented as the mean ± SD of n = 10. ^a, b^ and ^c^: Different subscript letters show statistical differences between treatments (*p* < 0.05). C_0_: extrapolated concentration without time 0; AUC0→ ∞: area under the curve from time zero to infinity; T_1/2_: elimination half-life; Vz: volume of distribution; Cl: clearance; MRT0 → ∞: mean residence time from zero to infinity.

**Table 2:** Pharmacokinetic parameters of O-desmethyltramadol (M1) after intravenous (IV) administration of tramadol (2 mg∙kg^−1^) in association with metamizole (10 mg∙kg^−1^ or 25 mg∙kg^−1^) in ten donkeys.

M1	(Tramadol 2 mg∙kg^−1^ IV) Mouta et al., 2021 [19]	T_2_M_10_ (Tramadol 2 mg∙kg^−1^ and Metamizole 10 mg∙kg^−1^ IV)	T_2_M_25_ (Tramadol 2 mg∙kg^−1^ and Metamizole 25 mg∙kg IV)
Cmax (ng/mL)	90 ± 61 ^a^	94 ± 22 ^a^	124 ± 30 ^b^
Tmax (h)	1.00 ± 0.16 ^a^	0.72 ± 0.14 ^a^	0.91 ± 0.08 ^a^
AUC0 → ∞ (h.ng/mL)	379 ± 238 ^a^	303 ± 76 ^a^	584 ± 412 ^b^
T_1/2_ (h)	8.43 ± 3.57 ^a^	6.11 ± 1.35 ^a^	13.50 ± 2.62 ^b^
Vz (L/h/kg)	NA	8.05 ± 5.12 ^a^	8.32 ± 7.73 ^a^
Cl (L/h/kg)	NA	9.32 ± 6.51 ^a^	5.48 ± 2.54 ^a^
MRT0 → ∞ (h)	10.80 ± 4.30 ^a^	5.09 ± 0.91 ^b^	18.25 ± 4.35 ^c^

Notes: Results are presented as the mean ± SD of n = 10. ^a, b^ and ^c^: different subscript letters show statistical differences between treatments (*p* < 0.05). Cmax: maximum concentration; Tmax: time to peak concentration; AUC0 → ∞: area under the curve from time zero to infinity; T_1/2_: elimination half-life; Vz: volume of distribution; Cl: clearance; MRT0 → ∞: mean residence time from zero to infinity; NA: not applicable.

**Table 3:** Pharmacokinetic parameters of 4-methylaminoantipyrine (4-MAA) after intravenous (IV) administration of tramadol (2 mg∙kg^−1^) in association with metamizole (10 mg∙kg^−1^ or 25 mg∙kg^−1^) in ten donkeys.

4-MAA	(Metamizol 10 mg∙kg^−1^ IV) Macêdo et al., 2021 [20]	T_2_M_10_ (Tramadol 2 mg∙kg^−1^ and Metamizol 10 mg∙kg^−1^ IV)	(Metamizol 25 mg∙kg^−1^ IV) Macêdo et al., 2021 [20]	T_2_M_25_ (Tramadol 2 mg∙kg^−1^ and Metamizol 25 mg∙kg^−1^ IV)
C_0_ (µg/mL)	31 ± 9.7 ^a^	109 ± 29 ^b^	100 ± 34 ^b^	128 ± 30 ^b^
AUC0 → ∞ (h.µg/mL)	14.51 ± 1.9 ^a^	53.70 ± 7.3 ^b^	44.78 ± 5.5 ^b^	52.2 ± 4.4 ^b^
T_1/2_ (h)	2.69 ± 0.34 ^a^	2.05 ± 0.19 ^a^	3.62 ± 0.24 ^b^	4.51 ± 0.94 ^b^
Vz (L/h/kg)	NA	1.6 ± 0.1 ^a^	NA	3.4 ± 0.2 ^b^
Cl (L/h/kg)	NA	5.0 ± 0.5	NA	4.9 ± 0.5
MRT0 → ∞ (h)	2.84 ± 0.3 ^a^	1.92 ± 0.24 ^a^	3.92 ± 0.36 ^b^	3.99 ± 0.74 ^b^

Notes: Results are presented as the mean ± SD of n = 10. ^a, b^: Different subscript letters show statistical differences between treatments (*p* < 0.05). C_0_: extrapolated concentration without time 0; AUC0 → ∞: area under the curve from time zero to infinity; T_1/2_: elimination half-life; Vz: volume of distribution; Cl: clearance; MRT0 → ∞: mean residence time from zero to infinity; NA: not applicable.

**Table 4:** Pharmacokinetic parameters of 4-aminoantipyrine (4-AA) after intravenous (IV) administration of tramadol (2 mg∙kg^−1^) in association with metamizole (10 mg∙kg^−1^ or 25 mg∙kg^−1^) in ten donkeys.

4-AA	(Metamizol 10 mg∙kg^−1^ IV) Macêdo et al., 2021 [20]	T_2_M_10_ (Tramadol 2 mg∙kg^−1^ and Metamizol 10 mg∙kg^−1^ IV)	(Metamizol 25 mg∙kg^−1^ IV) Macêdo et al., 2021 [20]	T_2_M_25_ (Tramadol 2 mg∙kg^−1^ and Metamizol 25 mg∙kg^−1^ IV)
Cmax (µg/mL)	1598 ± 0.25 ^a^	1941 ± 0.46 ^a^	2855 ± 0.55 ^b^	1067 ± 0.14 ^a^
Tmax (h)	0.22 ± 0.06 ^a^	1.56 ± 0.65 ^b^	0.15 ± 0.06 ^a^	0.91 ± 0.36 ^b^
AUC0→ ∞ (h.µg/mL)	6801 ± 1569 ^a^	14,175 ± 4367 ^b^	12,494 ± 1532 ^b^	11,981 ± 2583 ^b^
T_1/2_ (h)	6.37 ± 1.30 ^a^	9.41 ± 2.42 ^b^	7.11 ± 1.01 ^a^	10.47 ± 0.98 ^b^
Vz (L/h/kg)	NA	33.2 ± 3.4 ^a^	NA	37.1 ± 3.5 ^a^
Cl (L/h/kg)	NA	3.1 ± 0.3 ^a^	NA	2.5 ± 0.3 ^a^
MRT0 → ∞ (h)	10.95 ± 1.61 ^a^	14.04 ± 3.54 ^a^	11.20 ± 1.43 ^a^	15.55 ± 1.41 ^a^

Notes: Results are presented as the mean ± SD of n = 10. ^a, b^: different subscript letters show statistical differences between treatments (*p* < 0.05). Cmax: maximum concentration; Tmax: time to peak concentration; AUC0 → ∞: area under the curve from time zero to infinity; T_1/2_: elimination half-life; Vz: volume of distribution; Cl: clearance; MRT0 → ∞: mean residence time from zero to infinity; NA: not applicable.

## Data Availability

The data presented in this study are available on request from the corresponding author.
